# Family Bonds with Pets and Mental Health during COVID-19 in Australia: A Complex Picture

**DOI:** 10.3390/ijerph20075245

**Published:** 2023-03-23

**Authors:** Shannon K. Bennetts, Tiffani Howell, Sharinne Crawford, Fiona Burgemeister, Kylie Burke, Jan M. Nicholson

**Affiliations:** 1 Judith Lumley Centre, School of Nursing and Midwifery, La Trobe University, Bundoora, VIC 3086, Australia; 2 Intergenerational Health Group, Murdoch Children’s Research Institute, Parkville, VIC 3052, Australia; 3 Anthrozoology Research Group, School of Psychology and Public Health, La Trobe University, Bendigo, VIC 3552, Australia; 4 Metro North Mental Health, Metro North Health, Herston, QLD 4029, Australia

**Keywords:** pet-owner bonds, human-animal relationships, companion animals, child mental health, parent mental health, children, health promotion, mental health, COVID-19, Ottawa Charter

## Abstract

The COVID-19 pandemic has drawn attention to the health-promoting features of human-animal relationships, particularly for families with children. Despite this, the World Health Organization’s (1986) Ottawa Charter remains human-centric. Given the reciprocal health impacts of human-animal relationships, this paper aims to (i) describe perceived pet-related benefits, worries, and family activities; and to (ii) examine differences in perceived benefits, worries, and activities for parents and children with and without clinical mental health symptoms. We recruited 1034 Australian parents with a child < 18 years and a cat or dog via a national online survey between July and October 2020. Most parents reported their pet was helpful for their own (78%) and their child’s mental health (80%). Adjusted logistic regression revealed parents with clinical psychological distress were 2.5 times more likely to be worried about their pet’s care, well-being, and behaviour (OR = 2.56, *p* < 0.001). Clinically anxious children were almost twice as likely to live in a family who engages frequently in pet-related activities (e.g., cooked treats, taught tricks, OR = 1.82, *p* < 0.01). Mental health and perceived benefits of having a pet were not strongly associated. Data support re-framing the Ottawa Charter to encompass human-animal relationships, which is an often-neglected aspect of a socioecological approach to health.

## 1. Introduction

The COVID-19 pandemic has drawn greater attention to the role of family pets for promoting human health and well-being. This global public health emergency—associated with ‘stay at home’ orders across much of the world—coincided with record demand for pet adoptions [1] and significant increases in demand for child and adult mental health support services [2,3]. In Australia, pet ownership increased from 61% in 2019 to 69% in 2021 [4], with similar trends observed in the USA (increasing from 67% in 2019 to 70% in 2021) [5]. Around 75% of Australian children are now being raised alongside a pet [6], after what has been a “generation-defining” event which will have ongoing mental impacts for some young people [7]. As the World Health Organization asserts, “there is no health without mental health” [8]. We present here national evidence from Australian parents with children and pets to guide future health promotion efforts to support parent, child, and family mental health, considered with respect to the Ottawa Charter. 

The multidisciplinary study of interactions between human and non-human animals—“anthrozoology”—emerged in the late 1980s, following reports that human–pet interactions could have mental and physical health benefits [9]. In the decades since, a plethora of research has linked pets to increased physical activity, improved hormone levels and heart rate, non-judgemental companionship, and enhanced social skills in young children e.g., [6,10,11]. Despite this, evidence remains inconsistent, with a systematic review finding neutral, positive, and negative links between pets and human well-being [12]. Possible reasons for these inconsistencies include varied measurement (e.g., measuring presence/absence of a pet or the quality of human–pet bonds) and analytic approaches (e.g., whether potential confounding variables are controlled for). 

The COVID-19 pandemic presented a unique opportunity to understand how families with pets were living and coping and how pets might relate to parents’ and children’s mental health. For many families, stay-at-home orders to prevent viral transmission prompted strengthened, strained, or new relationships between children and their pets [13]. Spending time with pets has been reported as a coping strategy for stress during the pandemic among both children and adults [14,15]. With respect to mental health, there is growing evidence, including from our own work, that people with poor mental health, a lack of adequate (human) social supports, or poorer quality of life are more likely to report strong human–animal bonds compared to those with better self-reported health and well-being [15,16,17,18,19,20,21]. However, there remains a lack of evidence about how families view their pet’s role and how they choose to engage in pet-related activities—especially during times of stress and uncertainty—and how these might link with mental health. 

In light of the pandemic, we report here new evidence that underscores an urgent need to reconceptualise the 1986 Ottawa Charter [22] to incorporate the role of human–animal bonds for health promotion. According to the Ottawa Charter, health is defined as a “resource” and a “state of complete physical, mental and social well-being”. As part of the Charter, health promotion requires that people have control over their health to reach their potential. Since its inception, the Charter has been recognised as a guiding force in health promotion research and policy [23]. It acknowledges the inextricable links between people and their environments, upon which a socioecological approach to health is based. However, it remains human-centric and overlooks the role of animals for supporting and enhancing human well-being. Given that the Ottawa Charter focuses on health equity, we must consider the role of pets as a potentially critical support for people and communities experiencing adversity or poor well-being. This evidence, together with pandemic-related increases in pet acquisition and mental ill health, prompted our efforts to examine the relationships between mental health and families’ views and bonds with their pets. 

Using data from a national survey of Australian parents with a child < 18 years and a cat or dog, this paper aims to (i) describe perceived benefits of having a pet, worries about pets, and family engagement in pet-related activities during the COVID-19 pandemic; and to (ii) examine associations between clinically poor parent and child mental health and perceived benefits, worries, and family engagement in pet-related activities.

## 2. Materials and Methods

### 2.1. Design

Data for the *Parents, Pets & Pandemic Survey* were collected over a 12-week period, from July 2020 to October 2020, using an online cross-sectional design. Broadly, the survey aimed to capture a point-in-time ‘snapshot’ of the role of pets for families with children during a global pandemic. Eligibility criteria required that participants were: (i) over 18 years old; (ii) living in Australia; (iii) living with at least one child under 18 years at least some of the time; and (iv) living with at least one cat or dog. Ethical approval was granted by the La Trobe Human Research Ethics Committee (HEC20251). Participants were asked to tick a checkbox to indicate their electronic consent after being provided with an electronic copy of the Participant Information Statement. The survey was hosted by REDCap, which is a secure web-based data capture platform [24], and we also created a study Facebook page for recruitment purposes. Reporting on this survey was guided by the Checklist for Reporting Results of Internet E-Surveys (CHERRIES, [25]). 

#### 2.1.1. Context

Data collection coincided with the ‘second wave’ of COVID-19 in Australia, which most notably impacted the state of Victoria. By late October 2020 (the end of our data collection period), Australia had recorded 27,590 cases and 907 deaths. The largest number of national daily cases during the survey was 698 (5 August), and the largest number of daily deaths was 59 (4 September). Although these figures were low compared to many countries at the time, strict measures were implemented to minimise viral transmission. The 4.9 million residents of Melbourne, the capital of Victoria, were subject to a heavy lockdown between July and October 2020, during which there were only four permitted reasons to leave home: (i) essential work; (ii) purchasing essential supplies or services; (iii) restricted physical exercise for up to one hour per day; (iv) receiving or providing care. For part of this time, residents were also subject to a night-time curfew and could not move beyond 5 km away from their residence. The remaining Australian states and territories were experiencing low numbers of COVID-19 infections and were subject to some restrictions (e.g., masks, social distancing, limits on public and private gatherings with intermittent periods of lockdown similar to those in Victoria).

#### 2.1.2. Measures

A team of researchers with expertise in parenting, mental health, public health, and animal–human interaction selected the measures and tested the 15-min survey. Survey items were optional with the exception of key demographic items (e.g., gender, state). To avoid selection bias and minimise participant burden, participants with more than one child under 18 years were asked to respond about the child with the next birthday (‘focus child’). Participants with more than one cat or dog were asked to respond about the cat or dog who most recently joined the family (‘focus pet’). Although we acknowledge that human–pet attachments are likely to vary for different pets, this decision was made to avoid bias in pet selection, to minimise the burden of multi-pet responding, and to capture new pets who had been acquired during the pandemic. Participants were asked to enter the child’s and pet’s first name, initial, or a nickname. This information was auto-populated for all child- and pet-related items to ensure consistent reporting about the same ‘focus child’ and ‘focus pet’.

### 2.2. Benefits, Worries, and Pet-Related Activities

Parents responded to items about perceived family benefits of having a pet, worries related to the pet, and engagement in pet-related activities during the COVID-19 pandemic. A 5-item study-developed measure asked parents to rate the perceived benefits of having their pet, separately for parent and child mental and physical well-being, as well as for maintaining family routines. Each item was administered on a 5-point scale from 1 = very unhelpful to 5 = very helpful, producing a total possible score between 5 and 25, with higher scores indicating greater perceived benefit. Internal consistency in the current sample was good (*α* = 0.84).

A 5-item measure was created to evaluate parents’ worries, which was adapted from the *Pets in Australia Survey* [26]. Parents were asked: “During COVID-19, have you experienced any of the following worries because of having a cat or dog?” (i.e., worries about: caring for pet; pet’s behaviour; pet’s emotional well-being; adjusting to family routines). Items were administered on a 5-point scale from 0 = not at all to 4 = a lot, producing a total possible score between 0 and 20, with higher scores indicating more pet-related concerns. Internal consistency in the current sample was acceptable (*α* = 0.74).

Frequency of engagement in pet-related activities was captured using an adapted 13-item checklist from the *Pets in Australia Survey* [26]. Parents were asked: “How often have you/your family done the following with [*pet’s name*] during COVID-19?” on a 4-point scale from 0 = never to 3 = every day, producing a total possible score between 0 and 39, with higher scores indicating more engagement in pet-related activities (e.g., allowed them to sleep in/on the same bed as you or your child; cooked or made treats for them; created or posted content to a social media account for them). Internal consistency in the current sample was good (*α* = 0.82). 

In addition to these continuous variables, we created binary variables to capture high levels of perceived benefits, worries, and pet-related activities. This facilitated an analysis of associations between clinically poor mental health and perceived benefits, worries, and pet-related activities. To do this, we created dummy-coded variables based on 1 standard deviation above the mean for each variable, where 0 = low/average levels and 1 = high levels, equating to the top 17.2%, 16.8%, and 21.4%, respectively. See S2 for a list of survey measures used to capture perceived benefits, worries, and pet-related activities. 

### 2.3. Parent and Child Mental Health

Parent and child psychological well-being were assessed using brief validated measures. The K6 [27] is a six-item measure of psychological distress for adults administered on a 5-point scale from 1 = none of the time to 5 = all of the time, where higher scores indicate greater distress (e.g., “nervous”). Australian K6 scoring produces a possible score between 6 and 30, with a score of ≥19 indicative of a ‘probable serious mental illness’ (clinical range). Child anxiety was measured using four items adapted from the Spence Child Anxiety Scale (e.g., My child: “worries that something bad will happen to them”) [28,29] on a 4-point scale from 1 = never to 4 = always, producing a total score between 4 and 16. Permission was obtained from Professor Susan Spence for use of these four items for the current study. Child anxiety was selected as the most salient construct to briefly capture in a parent-reported survey during a time of uncertainty and change; however, this is only one aspect of children’s mental health (for example, it did not capture symptoms of low mood). In the absence of an established clinical cut-point for this adapted 4-item measure, we were guided by Sicouri and colleagues [30], who reported in 2020 that 20.2% of Australian children aged 4–17 years were experiencing anxiety symptoms in the clinical range (25-item parent-reported Revised Children’s Anxiety and Depression Scale). Applying this cut-point to the distribution of our child anxiety measure, children in the top approximately 20% were considered to be in the clinical range (i.e., total score of ≥9). Internal consistency in the current sample was good for both parent and child measures (*α* = 0.88 and *α* = 0.80, respectively). 

#### 2.3.1. Recruitment

Participants were recruited using both paid and unpaid Facebook advertising see [16] for further details. Briefly, unpaid advertising involved posting about the survey on Facebook pages or groups (e.g., pet, parent, or community groups and pages), while paid advertising involved Facebook campaigns conducted via the Facebook Ads Manager. We employed an active and flexible approach to recruitment, monitoring for ‘gaps’ in participant sub-groups (e.g., fathers), and adjusting recruitment strategies accordingly (e.g., running specific campaigns aimed at dads). This approach is effective and necessary for social media-based research recruitment [31]. At the end of the survey, participants could choose to enter a prize draw to receive one of ten AUD$20 gift cards. 

#### 2.3.2. Statistical Analyses

Analyses were conducted using StataSE Version 17 [32]. We firstly checked for evidence of multicollinearity using the *vif* command in Stata and checked histograms of the mental health and pet benefits, worries and activities variables for evidence of non-normality (See S1). 

To address Aim 1, we ran descriptive statistics to summarise perceived benefits of pets, worries about pets, and pet-related family activities (i.e., mean, range, and standard deviation for continuous variables, and number and percentage for categorical variables). This included examining total scores for benefits, worries, and activities for parents and children with clinically significant mental health symptoms. Independent sample *t*-tests were used to determine whether differences between clinical and non-clinical groups were statistically significant. 

To address Aim 2, we conducted unadjusted and adjusted logistic regression models separately to predict associations between poor mental health (independent variables: parent, child) and benefits, worries, and activities (dependent variables). Adjusted models controlled for ten variables known to be associated with parent and child well-being or human–pet bonds: parent gender, child gender, only child status, parent age, child age group (0–4; 5–9; 10–14; 15–17 years), parent education (with or without tertiary education), single parent, non-English speaking background, pet type (dog/cat), and neighbourhood disadvantage. Neighbourhood-level disadvantage was measured using the Index of Relative Socio-economic Disadvantage, which is based on postcode of residence [33]. Given the strong association between parent and child mental health [34], the parent models were adjusted for child mental anxiety, and the child models were adjusted for parent mental health. 

## 3. Results

### 3.1. Sample Descriptive

Participants ranged from 20 to 65 years of age (mean = 43 years, SD = 7 years); most were female (78%) and had a tertiary qualification (62%). Our sample included 16% single parents, 7% who spoke a non-English language at home, and 1% who identified as Indigenous. Focus children (those with the next birthday) represented a range of ages, with the largest group aged 10–14 years (38%). Focus child gender was roughly half female and male, and one-third were the family’s only child. For nearly two-thirds of respondents (65%), the focus pet (most recently acquired cat or dog) was a dog. Almost two-thirds of families (62%) were living in Victoria, and the remaining third were based in other Australian states and territories. Half (50%) were living in a metropolitan location in Australia. On average, participants were living in slightly more advantaged neighbourhoods compared to the Australian norm of 1000 (mean = 1022, SD = 59, range 838–1128). One-fifth (20%) had introduced a new cat or dog to the family during the COVID-19 pandemic. 

### 3.2. Aim 1: Perceived Benefits, Worries and Pet-Related Activities during COVID-19

Full sample item-level descriptives for benefits, worries, and pet-related activities are shown in Table 1, including the proportion of respondents who rated their pets as quite or very helpful (i.e., scores of 4 or 5 on a scale from 1–5), worried about their pet a bit or a lot (i.e., scores of 3 or 4 on a scale from 0–4), and engaged in pet-related activities often or every day (i.e., scores of 2 or 3 on a scale from 0–3). Around 80% of participants rated their pet as quite or very helpful for their child’s and their own mental health. Overall, the level of worry about pets was low; the most commonly reported concern was about the pet’s emotional well-being (8.2%). More than 80% talked to their pet as if they understood ‘often’ or ‘every day’, referred to themselves as the pet’s ‘parent’ (72.9%) and gave them premium or expensive food (62%). 

### 3.3. Aim 2: Associations between Poor Parent and Child Mental Health and Perceived Benefits, Worries and Pet-Related Activities

At the time of participation, 13.7% of parents were scoring in the clinical range for psychological distress (i.e., scores of ≥19 on a scale from 6–30). Guided by previous evidence [30] of around 20% prevalence for clinical child anxiety during mid-2020, children scoring ≥9 (20.1%) were classified as having anxiety in the clinical range. As shown in Table 2, parents experiencing psychological distress were more likely to have an anxious child during COVID-19, to be worried about their pet, and to be engaging in more pet-related activities. The strongest relationship was between perceived benefits of pet ownership and engagement in pet-related activities. That is, parents who reported greater benefits of having a pet were engaging in a broader range of pet-related activities more frequently. 

As shown in Table 3, for perceived benefits, differences between clinical and non-clinical groups were small or negligible (i.e., the association between greater perceived benefits and child anxiety was statistically significant, but the effect size was small and is unlikely to be large enough to be considered clinically relevant). Both parents and children with mental health symptoms in the clinical range reported greater (parent-reported) worry about their pet (moderate effect sizes). Similarly, parents and children in the clinical group engaged in more frequent pet-related activities compared to those in the non-clinical group (small to moderate effect sizes). 

Item-level descriptives are presented in Table 4 separately for clinical and non-clinical groups. Overall, parents reported that pets were very beneficial for their own and their child’s mental and physical health and for maintaining family routines. Mean scores ranged from 3.7 to 4.4 (scale 1–5), indicating parents on average thought their pets were “4 = quite” or “5 = very helpful”. The highest benefits ratings were for child mental health for children in the clinical group (M = 4.4, SD = 0.7, moderate effect size). When asked about concerns about their pet’s care, behaviour, or well-being, most parents were worried “0 = not at all” or “1 = a little” (mean scores ranged from 0.3 to 1.4 on a 0–5 scale). The greatest concern was about pet care (e.g., paying for food, accessing vet care) reported by parents in the clinical group (M = 1.4, SD = 1.3, large effect size). Pet-related activities that parents reported engaging in “2 = often” or “3 = every day” (scale 0–3) during the pandemic were talking to pets as if they understood (M_range_ = 2.3–2.5, SD_range_ = 0.8–0.9). Parents and children in the clinical range also “often” referred to themselves as the pet’s ‘parent’, allowed the pet to sleep in their bed, and gave them premium or expensive food (M_range_ = 2.0–2.3, SD_range_ = 1.0–1.2). 

Unadjusted and adjusted logistic regression analyses are presented in Table 5. Associations attenuated slightly after controlling for known demographic covariates, including parent or child mental health. Parents in the clinical range for psychological distress had 2.5 times the odds of having high worries about their pet’s care, behaviour, and well-being. Clinically anxious children had 1.8 times the odds of living in a family with high engagement in pet-related activities. 

## 4. Discussion

We describe here findings from a relatively large national study of Australian parents with children and pets during the COVID-19 pandemic between July and October 2020, which we consider in the context of the Ottawa Charter for health promotion. During this time of global change and uncertainty, we report on parents’ perceptions of pet-related benefits, worries and activities, including group differences for parents and children with or without mental health symptoms in the clinical range. Taken together, our findings contribute to a growing body of robust evidence that highlights the perceived importance of pets for health promotion, especially for those who may be vulnerable to mental ill health. 

Firstly, almost 14% of parents in our sample were experiencing clinical levels of psychological distress compared to estimates of around 6% in population-based studies prior to the COVID-19 pandemic [35]. Similarly, rates of anxiety disorders in Australian children prior to the pandemic were around 6–7% [36], indicating that that pandemic has prompted at least a three-fold increase in parent-reported child symptomatology (around 20% [30]). Parents consistently rated the benefits from their pets positively, for their own and their child’s mental and physical health, and for family routines. This echoes previous research, including our own qualitative research from this sample, about how pets can offer comfort, companionship, laughter, distraction, structure and routine [15,37], during a time of global uncertainty and disruption. Despite relatively low rates of COVID-19-related cases and deaths in Australia compared to many other countries, the importance of pets for human mental health during the pandemic has been widely reported across the world e.g., [1,18,38]. 

Pet-related worries were low overall; however, parents in the clinical range for psychological distress were more than twice as likely to feel worried about their pet compared to those in the non-clinical range (large effect size, ES = 0.84). Most commonly, this included concerns about caring for the pet, such as paying for food and accessing vet care, followed by concerns about the pet’s emotional well-being and concerns about the pet adjusting to household changes. Worries about accessing and affording timely veterinary care, food, and other supplies and services during the pandemic have been well-documented [38]. Indeed, financial concerns were exacerbated by pandemic restrictions, highlighting both access and equity issues which disproportionately affected vulnerable people and communities [39], such as those at risk of poor mental health. Previous research has found that highly empathetic people are more likely to have high anxiety [40], so it is possible that this anxiety might extend to worry about one’s pet. In line with recent findings from other studies [17,18,19], high levels of empathy toward animals often leads to the formation of very strong human–animal bonds (for example, evidenced by perceived benefits and worries about pets, and engagement in pet-related activities), and this often occurs alongside psychological vulnerability. For example, symptoms of anxiety, depression or other forms of psychological distress can prompt us to gravitate to pets for additional companionship and comfort, especially if we feel that existing human social supports are lacking or insufficient (as was the case during the COVID-19 ‘lockdowns’ [41]). Our finding lends further support to existing evidence that people who are experiencing psychological distress are more likely to form strong human–animal bonds, and that additional human supports might be warranted (e.g., clinical services). 

Considering strengthening evidence on how parents and children perceive and interact with their pets and their mental health, we advocate for considering revisions to the 1986 Ottawa Charter on health promotion to incorporate the significant influence of human-animal relationships. The Charter identifies five action areas for health promotion: (i) *Building healthy public policy*; (ii) *Creating supportive environments*; (iii) *Strengthening community action*; (iv) *Developing personal skills*; and (v) *Re-orienting health care services toward prevention of illness and promotion of health*. We suggest that human–animal bonds most closely align with areas 2 and 4. 

Firstly, the Ottawa Charter is grounded in socioecological theory; pets fit within the interpersonal level, as beings within the individual’s immediate sphere of influence. This is important because almost 70% of Australians now live with a pet [4], and most view their pet as a family member [26]. As our findings show, families engaged frequently in a very broad range of pet-related activities during the pandemic, from sleeping with their pet, to making pet treats, to sharing pet photos on social media, and re-arranging personal commitments around the pet’s needs. Furthermore, around 80% reported that their pet was helpful for their own mental health and their child’s mental health. These data demonstrate that pets are perceived as integral to the family unit. There was only modest evidence for links between mental health and perceived benefits of pets in our sample, possibly reflecting high overall scores. 

Secondly, the Charter seeks to guide actions that improve health equity. Our findings contribute to growing evidence linking psychological vulnerability with strong pet bonds [16,17,18,19], which might suggest that relevant and accessible human supports are lacking. For example, Lass-Hennemann and colleagues [19] recently found that attachment to humans mediated the positive association between pet attachment and poor mental health; that is, strong pet bonds were associated with more insecure human attachment styles (i.e., less trust in other people, more fear of rejection). Thus, pets may have an important role to play in health promotion and ensuring health equity. Re-imagining the Charter to encompass animals could include how they can facilitate the creation of supportive environments (action area 2) and personal skill development (action area 4). Our ways of living and working should consider the health of both people and animals, as these are inextricably linked. 

### Limitations and Future Research

We acknowledge that these data are cross-sectional and we therefore cannot imply causality with respect to mental health, views about pets, and engagement in pet-related activities. Longitudinal evidence is required to delineate the mechanisms with which pets support or detract from human mental health as well as how human–pet bonds relate to pet behaviour and well-being. Furthermore, our data are parent-reported and may therefore vary from children’s views of their own anxiety [42], views about pet benefits and worries, and perceptions about family engagement in pet-related activities. We measured child anxiety specifically, as this was considered the most salient construct to briefly capture in a parent survey, but we did not capture other potentially important aspects of child mental health such as low mood. Participants reported on the cat or dog who most recently joined their family to avoid self-selection bias and to reduce the burden of reporting on multiple pets. However, benefits, worries and activities might vary depending on the pet of focus. It is also possible that parents’ views in the current study may not be representative of all Australian families with a cat or dog, given that they responded to advertisements about a pet-related survey. As is typical with survey-based parenting research [43], male participants were under-represented (22%); however, proportions of tertiary-educated respondents (62%) and single parents (16%) were close to national population-based estimates [44,45]. 

## 5. Conclusions

Our findings extend previous research on the role of pets for human mental health, providing a national ‘snapshot’ of how Australian families with pets were experiencing the COVID-19 pandemic. The strong link between poor parent mental health and greater pet-related worries speaks to the critical role of pets, especially for those who may be at risk of poorer health and well-being. Longitudinal research is required to examine the mechanisms underlying this link between strong human–pet bonds and poor mental health, given that the majority of existing research has been cross-sectional. 

There are opportunities for enhancing personal and social development by considering the role of animals at different life stages and facilitating healthy human–animal interactions at home, school, work, and other community settings. This could include the introduction of policies and supports to accommodate pets in workplaces, schools, or clinical environments, particularly for adults, adolescents or children with mental health challenges and a strong bond with their pet. For people with poor mental health, the presence of their pet may contribute to more supportive environments and facilitate greater social connectedness. However, the appropriateness of animals accompanying humans outside the home environment must be considered with respect to the animal’s temperament and observed preferences. Lastly, there are opportunities to further our understanding of how human–pet interactions can enhance the health of both human and pets–including both physical and mental wellbeing. With three-quarters of Australian children now raised alongside pets, investigating how we can nurture mutually health-promoting child–pet interactions will be an important next step. An Ottawa Charter that acknowledges the role of animals in health promotion will provide the foundations for this important ongoing work. 

## Figures and Tables

**Table 1:** Full sample item-level descriptives for perceived pet benefits, worries about pet, and pet-related activities (N = 1034).

	N (%)
**Benefits of Pet (possible score 1–5)**	**Quite or Very Helpful**
Your own mental well-being	809 (78.2)
Child’s mental well-being	828 (80.2)
Your physical well-being	624 (60.3)
Child’s physical well-being	582 (56.3)
Maintaining family routines	677 (65.5)
**Worries about Pet (possible score** **0–4)**	**Quite a Bit or a Lot of Worry**
Worries about caring for pet (e.g., paying for food, accessing vet care).	67 (6.5)
Worries about interactions between pet and child	26 (2.5)
Worries about pet’s behaviour (e.g., biting, scratching, barking)	61 (5.9)
Worries about your pet’s emotional well-being (e.g., seems unsettled or anxious)	85 (8.2)
Worries about how pet is adjusting to changes in your family routines	77 (7.5)
**Pet-Related Activities (possible score 0–3)**	**Often or Every Day**
Talked to them as if they understand you	848 (82.2)
Allowed them to sleep in/on the same bed as you or your child	632 (61.2)
Referred to yourself as their ‘parent’	751 (72.9)
Given them treats or new toys	579 (56.1)
Cooked or made treats for them	301 (29.3)
Left on the heating/cooling, lights, or TV/radio for them	405 (39.2)
Rearranged personal commitments around them	296 (28.7)
Taught them tricks or trained them to do something	359 (34.9)
Given them premium/expensive pet food or human food	641 (62.1)
Participated in cat/dog groups or pages on social media	417 (40.6)
Created or posted content to a social media account for them	246 (23.9)
Dressed them in outfits/costumes	50 (4.9)
Worn matching outfits/accessories with them	5 (0.5)

Note: denominators range from 1027 to 1034 due to missing data on some items. Items presented in the order in which they were administered.

**Table 2:** Correlations between total scores for benefits, worries, activities, and parent and child mental health (all continuous variables, N = 1034).

	1	2	3	4	5
1. Parent Mental Health	-	0.34 ***	0.07 *	0.33 ***	0.11 ***
2. Child Anxiety		-	0.14 ***	0.23 ***	0.15 ***
3. Benefits			-	0.05	0.47 ***
4. Worries				-	0.21 ***
5. Pet-Related Activities					-

Note: spearman’s coefficients reported due to non-normal distribution of benefits, worries, parent and child anxiety. Bold = significant, * *p* < 0.05, *** *p* < 0.001.

**Table 3:** Summary descriptives for perceived benefits, worries and pet-related activities for overall sample and for parents and children with poor mental health (N = 1034).

	Full Sample	Parent			Child		
		Non-Clinical (N = 892)	Clinical (N = 140)	*p* (ES)	Non-Clinical (N = 826)	Clinical (N = 208)	*p* (ES)
**Parent Mental Health**, M (SD), possible score: 6–30	12.8 (5.1)	11.3 (3.5)	22.2 (2.8)	-	12.1 (4.7)	15.3 (5.5)	-
**Child Anxiety**, M (SD), possible score: 4–16	6.8 (2.4)	6.6 (2.2)	8.4 (2.9)	-	5.8 (1.3)	10.7 (1.8)	-
**Total Benefits**, M (SD) possible score: 5–25	19.7 (3.3)	19.7 (3.3)	20.0 (3.6)	0.32 (0.09)	19.6 (3.3)	20.3 (3.3)	**<0.01 (0.23)**
**Total Worries**, M (SD) possible score: 0–20	3.2 (3.3)	2.9 (3.1)	5.1 (4.0)	**<0.001 (0.66)**	2.9 (3.2)	4.3 (3.7)	**<0.001 (0.43)**
**Total Activities**, M (SD), possible score: 0–39	16.6 (7.1)	16.3 (6.9)	18.5 (7.8)	**<0.001 (0.31)**	16.1 (7.0)	18.7 (7.1)	**<0.001 (0.37)**

Parents with probable mental illness defined by K6 scores ≥19. Children with clinically significant anxiety defined by scores ≥9. Two-sample *t*-test between clinical and non-clinical groups. Bold = significant. ES = effect size (mean difference divided by standard deviation).

**Table 4:** Item-level descriptives for perceived pet benefits, worries about pet, and pet-related activities separately for parent and child non-clinical and clinical mental health groups (N = 1034).

	Parent Mental Health			Child Anxiety		
	Non-Clinical (N = 892)	Clinical (N = 140)	*p* (ES)	Non-Clinical (N = 826)	Clinical (N = 208)	*p* (ES)
**Benefits of Pet, M (SD), possible score: 1–5**						
Your own mental well-being	4.1 (0.8)	4.2 (0.9)	0.39 (0.08)	4.1 (0.8)	4.2 (0.8)	**0.005 (0.22)**
Child’s mental well-being	4.2 (0.8)	4.3 (0.8)	0.10 (0.15)	4.1 (0.8)	4.4 (0.7)	**<0.001 (0.39)**
Your physical well-being	3.8 (0.9)	3.8 (1.0)	0.96 (0.00)	3.8 (0.9)	3.9 (1.0)	0.39 (0.07)
Child’s physical well-being	3.7 (0.9)	3.8 (1.0)	0.31 (0.09)	3.7 (0.9)	3.9 (0.9)	**0.003 (0.23)**
Maintaining family routines	3.9 (0.9)	3.9 (0.9)	0.72 (0.03)	3.9 (0.9)	3.9 (0.9)	0.96 (0.00)
**Worries about Pet, M (SD) (possible score 0–4)**						
Worries about caring for pet (e.g., paying for food, accessing vet care).	0.6 (0.9)	1.4 (1.3)	**<0.001 (0.84)**	0.6 (0.9)	1.0 (1.1)	**<0.001 (0.39)**
Worries about interactions between pet and child	0.3 (0.7)	0.6 (0.9)	**<0.001 (0.42)**	0.3 (0.7)	0.4 (0.8)	**0.049 (0.15)**
Worries about pet’s behaviour (e.g., biting, scratching, barking)	0.6 (0.9)	0.8 (1.1)	**0.012 (0.23)**	0.6 (0.9)	0.8 (1.0)	**0.02 (0.18)**
Worries about your pet’s emotional well-being (e.g., seems unsettled or anxious)	0.7 (1.0)	1.1 (1.2)	**<0.001 (0.39)**	0.7 (1.0)	1.1 (1.2)	**<0.001 (0.43)**
Worries about how pet is adjusting to changes in your family routines	0.7 (1.0)	1.1 (1.2)	**<0.001 (0.44)**	0.7 (1.0)	1.0 (1.2)	**<0.001 (0.30)**
**Pet-Related Activities, M (SD) (possible score 0–3)**						
Talked to them as if they understand you	2.4 (0.9)	2.4 (0.8)	0.43 (0.07)	2.3 (0.9)	2.5 (0.8)	**0.014 (0.19)**
Allowed them to sleep in/on the same bed as you or your child	1.8 (1.3)	2.0 (1.2)	**0.04 (0.18)**	1.8 (1.3)	2.0 (1.3)	0.08 (0.14)
Referred to yourself as their ‘parent’	2.1 (1.1)	2.2 (1.2)	0.46 (0.07)	2.1 (1.1)	2.3 (1.0)	**0.008 (0.21)**
Given them treats or new toys	1.7 (0.9)	1.7 (1.0)	0.89 (0.01)	1.6 (0.9)	1.8 (0.9)	**0.007 (0.21)**
Cooked or made treats for them	1.0 (1.0)	1.2 (1.1)	**0.03 (0.20)**	0.9 (1.0)	1.2 (1.1)	**0.006 (0.22)**
Left on the heating/cooling, lights, or TV/radio for them	1.2 (1.1)	1.5 (1.2)	**<0.001 (0.31)**	1.2 (1.1)	1.4 (1.1)	**0.001 (0.25)**
Rearranged personal commitments around them	1.0 (1.0)	1.3 (1.0)	**0.004 (0.26)**	1.0 (0.9)	1.2 (1.1)	**<0.001 (0.26)**
Taught them tricks or trained them to do something	1.1 (1.0)	1.3 (1.1)	**0.03 (0.19)**	1.1 (1.0)	1.3 (1.0)	**0.007 (0.21)**
Given them premium/expensive pet food or human food	1.8 (1.1)	2.1 (1.0)	**0.01 (0.23)**	1.8 (1.1)	2.1 (1.0)	**0.003 (0.23)**
Participated in cat/dog groups or pages on social media	1.2 (1.2)	1.4 (1.2)	0.25 (0.10)	1.2 (1.2)	1.5 (1.2)	**0.006 (0.21)**
Created or posted content to a social media account for them	0.7 (1.0)	0.9 (1.1)	**0.04 (0.19)**	0.7 (1.0)	1.0 (1.1)	**<0.001 (0.27)**
Dressed them in outfits/costumes	0.2 (0.5)	0.5 (0.8)	**<0.001 (0.42)**	0.2 (0.5)	0.4 (0.8)	**0.002 (0.24)**
Worn matching outfits/accessories with them	0.03 (0.2)	0.1 (0.3)	0.12 (0.14)	0.03 (0.2)	0.1 (0.3)	**0.012 (0.20)**

Bold = significant. ES = effect size (mean difference divided by standard deviation). Two-sample *t*-test between clinical and non-clinical groups.

**Table 5:** Unadjusted and adjusted logistic regression models examining associations between poor parent and child mental health and high ratings of pet benefits, worries and activities.

	Model 1: High Benefits		Model 2: High Worries		Model 3: High Activities	
	Unadj OR (95% CI)	Adj OR (95% CI)	Unadj OR (95% CI)	Adj OR (95% CI)	Unadj OR (95% CI)	Adj OR (95% CI)
**Parent: Clinical Range**	1.24 (0.79, 1.94)	1.05 (0.64, 1.73)	3.20 *** (2.15, 4.76)	2.56 *** (1.64, 3.99)	1.65 * (1.11, 2.46)	1.32 (0.85, 2.06)
**Child: Clinical Range**	1.61 * (1.11, 2.33)	1.50 (0.996, 2.26)	1.99 *** (1.38, 2.87)	1.40 (0.92, 2.14)	2.07 *** (1.48, 2.91)	1.82 ** (1.25, 2.66)

Note: Unadj = unadjusted; Adj = adjusted; OR = Odds Ratio; CI = Confidence Interval. * *p* < 0.05, ** *p* < 0.01, *** *p* < 0.001; Bold = significant. Each model controlled for parent and child gender, child without siblings, parent age, child age group, parent education, single parent, language other than English spoken at home, pet type (cat/dog), and neighbourhood disadvantage. Parent model controls for child anxiety and child model control for parent mental health.

## Data Availability

Data presented in this paper is not publicly available because participants only provided consent for the purpose of the current study.

## Supplementary Materials

The following supporting information can be downloaded at: https://www.mdpi.com/article/10.3390/ijerph20075245/s1, S1: Histograms for parent psychological distress, child anxiety, pet benefits, pet worries, and pet-related activities; S2: Survey measures for pet benefits, pet worries, and pet-related activities.
