# Supplementary material for: Family Bonds with Pets and Mental Health during COVID-19 in Australia: A Complex Picture
Source: Int J Environ Res Public Health. 2023 Mar 23;20(7):5245. doi: 10.3390/ijerph20075245 (PMC10094414; doi:10.3390/ijerph20075245)
Supplement: Supplementary file 1 [file ijerph-20-05245-s001.zip › ijerph-2262861-supplementary.pdf]

S1. Histograms for parent psychological distress, child anxiety, pet benefits, pet worries, and pet-related activities.

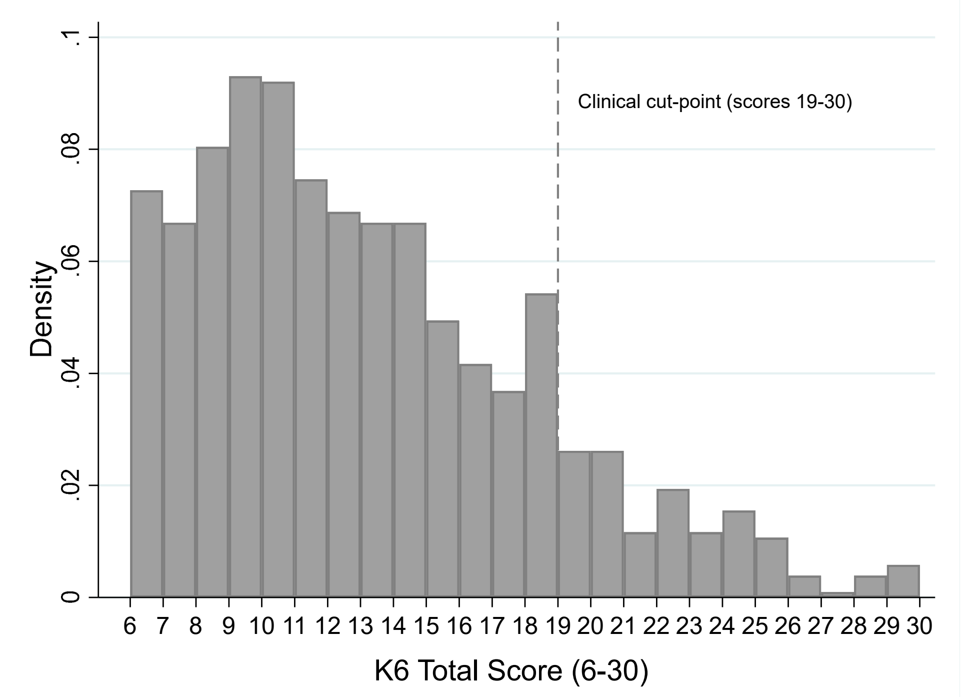

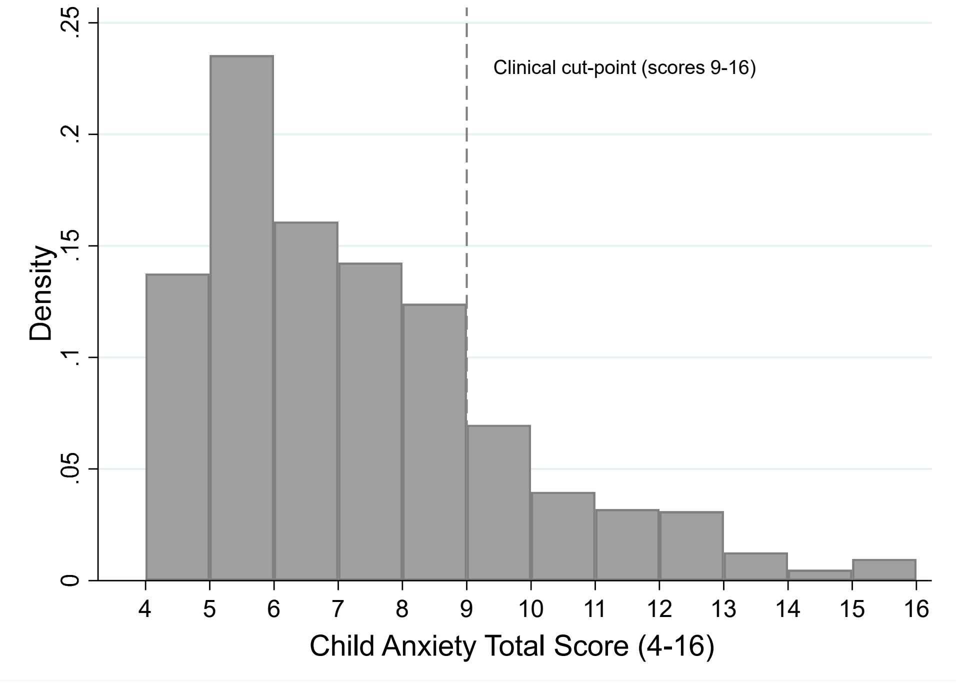

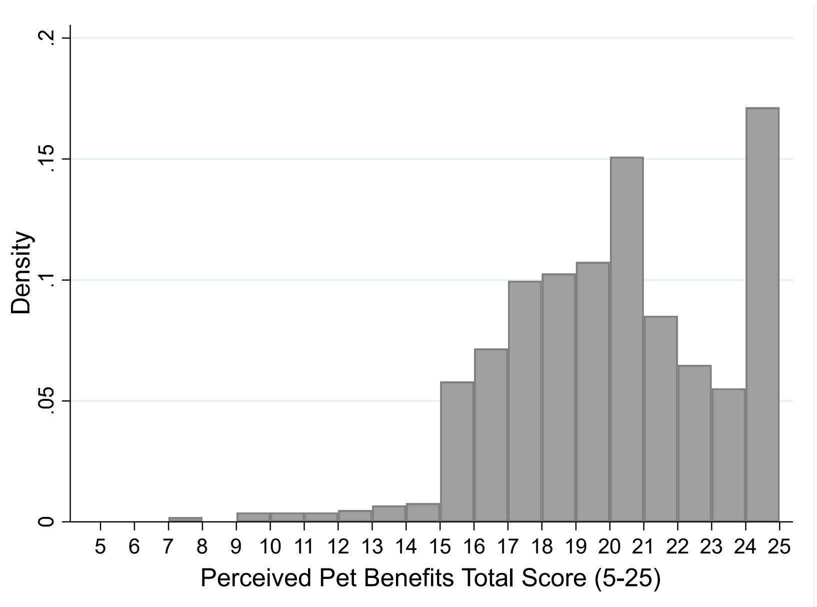
Density
0
.05
.1
.15
.2
5 6 7 8 9 10 11 12 13 14 15 16 17 18 19 20 21 22 23 24 25
Perceived Pet Benefits Total Score (5-25)

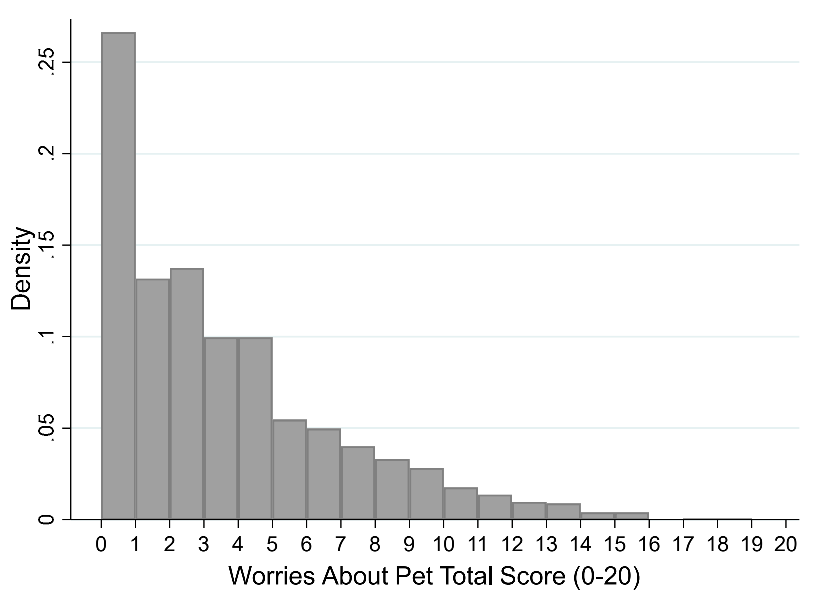
Density
0
.05
.1
.15
.2
.25
0 1 2 3 4 5 6 7 8 9 10 11 12 13 14 15 16 17 18 19 20
Worries About Pet Total Score (0-20)

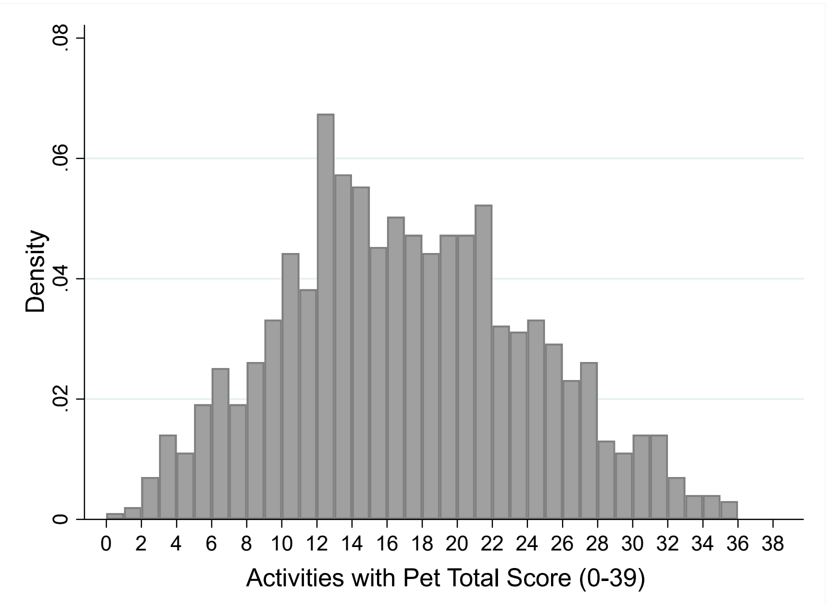
Density
0
.02
.04
.06
.08
0 2 4 6 8 10 12 14 16 18 20 22 24 26 28 30 32 34 36 38
Activities with Pet Total Score (0-39)

S2. Survey measures for pet benefits, pet worries, and pet-related activities.

During COVID-19, how helpful has [pet] been for...?

		Very un-helpful	Quite un-helpful	Neither helpful or unhelpful	Quite helpful	Very help-ful
a.	Your own mental wellbeing	☐1	☐2	☐3	☐4	☐5
b.	[child]'s mental wellbeing	☐1	☐2	☐3	☐4	☐5
c.	Your physical wellbeing	☐1	☐2	☐3	☐4	☐5
d.	[child]'s physical wellbeing	☐1	☐2	☐3	☐4	☐5
e.	Maintaining family routines	☐1	☐2	☐3	☐4	☐5

Source: study-developed.

During COVID-19, have you experienced any of the following worries because of having a cat or dog?

		Not at all	A little	Some	Quite a bit	A lot
a.	Worries about caring for [pet] (e.g., paying for food, accessing vet care).	☐0	☐1	☐2	☐3	☐4
b.	Worries about interactions between [pet] and [child]	☐0	☐1	☐2	☐3	☐4
c.	Worries about [pet]'s behaviour (e.g., biting, scratching, barking)	☐0	☐1	☐2	☐3	☐4
d.	Worries about your [pet]'s emotional wellbeing (e.g., seems un-settled or anxious)	☐0	☐1	☐2	☐3	☐4
e.	Worries about how [pet] is adjusting to changes in your family routines	☐0	☐1	☐2	☐3	☐4

Source: adapted from items featured in the Pets in Australia Survey (Animal Medicines Australia, 2019).

How often have <u>you/your family</u> done the following with [pet] during COVID-19?

		Never	Sometimes	Often	Every Day
a.	Talked to them as if they understand you	☐0	☐1	☐2	☐3
b.	Allowed them to sleep in/on the same bed as you or your child	☐0	☐2	☐2	☐3
c.	Referred to yourself as their 'parent'	☐0	☐1	☐2	☐3
d.	Given them treats or new toys	☐0	☐1	☐2	☐3
e.	Cooked or made treats for them	☐0	☐1	☐2	☐3
f.	Left on the heating/cooling, lights, or TV/radio for them	☐0	☐1	☐2	☐3
g.	Rearranged personal commitments around them	☐0	☐1	☐2	☐3
h.	Taught them tricks or trained them to do something	☐0	☐1	☐2	☐3
i.	Given them premium/expensive pet food or human food	☐0	☐1	☐2	☐3
j.	Participated in cat/dog groups or pages on social media	☐0	☐1	☐2	☐3
k.	Created or posted content to a social media account for them	☐0	☐1	☐2	☐3
l.	Dressed them in outfits/costumes	☐0	☐1	☐2	☐3
m.	Worn matching outfits/accessories with them	☐0	☐1	☐2	☐3

Source: adapted from items featured in the Pets in Australia Survey (Animal Medicines Australia, 2019).
